# Halobenzene Clathrates of the Porous Metal–Organic Spin-Crossover Framework [Fe(tvp)_2_(NCS)_2_]_*n*_. Stabilization of a Four-Step Transition

**DOI:** 10.1021/acs.inorgchem.2c00014

**Published:** 2022-03-02

**Authors:** Alejandro Orellana-Silla, Francisco Javier Valverde-Muñoz, M. Carmen Muñoz, Carlos Bartual-Murgui, Sacramento Ferrer, José Antonio Real

**Affiliations:** † Instituto de Ciencia Molecular/Departamento de Química Inorgánica, Universidad de Valencia, 46980 Paterna, Valencia, Spain; ‡ Departamento de Física Aplicada, Universitat Politècnica de València, 46022 Valencia, Spain; § Departamento de Química Inorgánica, Universidad de Valencia, 46100 Burjassot, Valencia,Spain

## Abstract

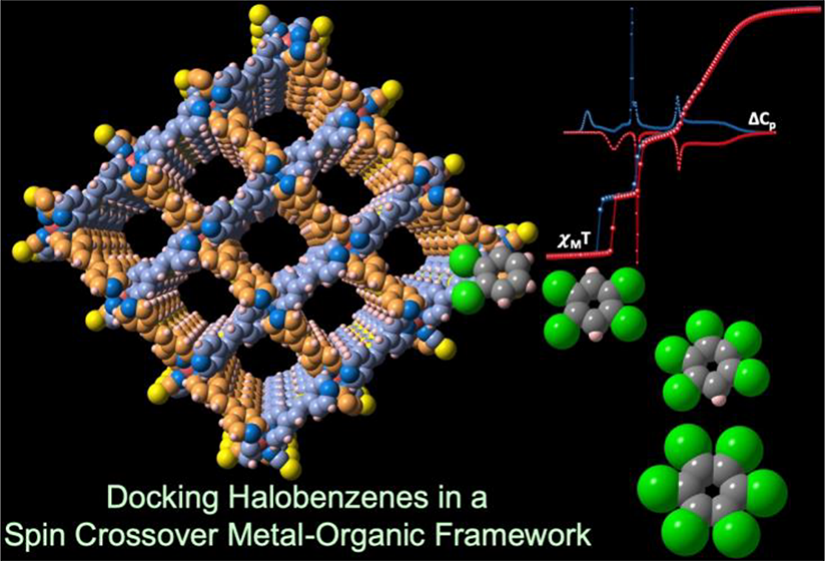

Here we show that the porous metal–organic spin crossover (SCO) framework [Fe(tvp)_2_(NCS)_2_]@4(CH_3_CN·H_2_O) [**1@4(CH**_**3**_**CN·H**_**2**_**O)**] is an excellent precursor material for the systematic synthesis, via single-crystal to single-crystal transformation, of a series of halobenzene clathrates. Immersion of samples constituted of single crystals of **1@4(CH**_**3**_**CN·H**_**2**_**O)** in the liquid halobenzenes PhX_*n*_, X = F (*n* = 1–6), X = Cl (*n* = 1, 2), and X = Br (*n* = 1) at room temperature induces complete replacement of the guest molecules by PhX_*n*_ to afford **1@2PhX**_***n***_. Single-crystal analyses of the new clathrates confirm the integrity of the porous framework with the PhX_*n*_ guests being organized by pairs via π-stacking filling the nanochannels. The magnetic and calorimetric data confirm the occurrence of practically complete SCO behavior in all of the clathrates. The characteristic SCO equilibrium temperatures, *T*_1/2_, seem to be the result of a subtle balance in the host–guest interactions, which are temperature- and spin-state-dependent. The radically distinct supramolecular organization of the PhCl_2_ guests in **1@2PhCl**_**2**_ affords a rare example of four-step SCO behavior following the sequence [HS_1_:LS_0_] ↔ [HS_2/3_:LS_1/3_] ↔ [H*S*_1/2_:L*S*_1/2_] ↔ [HS_1/4_:LS_3/4_] ↔ [HS_0_:LS_1_], which has been structurally characterized.

## Abstract 2

Guest exchange via single-crystal to single-crystal transformation has been revealed to be an excellent approach to systematically stow halobenzene molecules in the nanochannels of the porous spin-crossover (SCO) metal−organic framework [Fe^II^(tvp)_2_(NCS)_2_]_*n*_. This has enabled the unveiling of key structural factors leading to a four-step SCO behavior.

## Introduction

Octahedral Fe^II^ spin-crossover complexes are a singular class of electronically labile materials reversibly switching between the high (HS, t_2g_^4^e_g_^2^) and low (LS, t_2g_^6^e_g_^0^) spin states in response to temperature, pressure, light irradiation, and interaction with guest molecules. The HS ↔ LS switch is accompanied by noticeable changes in the magnetic, electrical, and optical properties of the material. Furthermore, due to the antibonding nature of the e_g_ orbitals, their population–depopulation is strongly coupled to structural changes involving metal to ligand bond lengths and angles, resulting in an expansion–contraction of the coordination octahedron. In the crystalline state, the change in size of the Fe^II^ octahedron is the source of the elastic stress responsible for cooperativity, which critically depends on the coupling between the Fe^II^ centers mediated by intermolecular interactions.^1−9^ Strong coupling favors abrupt HS ↔ LS transitions accompanied in favorable cases by hysteretic behavior, which confers a memory effect to the SCO material. In absence of antagonistic elastic stresses in the crystal, the HS ↔ LS transition occurs in one step while the presence of elastic frustration^10−12^ divides the HS ↔ LS transformation into successive steps (two, three, or more) as a mechanism to minimize the energy cost of the transformation, thereby conferring multistability (three or more steps) to the material.^13−23^ The possibility of controlling the HS ↔ LS switching at macro-, micro-, and nanometric levels has created important possibilities for technological applications.^24−26^

Among the Fe^II^ SCO complexes, coordination polymers have long attracted much interest because they represent an excellent opportunity to explore different strategies for coupling SCO centers and thus understand the mechanisms that control the cooperativity.^6,27−33^ This interest was significantly increased with the first porous SCO coordination polymers reported, since they additionally afforded the prospect of investigating the impact of host–guest interactions on the SCO properties. In this context, the family of doubly interpenetrated two-dimensional Fe^II^ SCO coordination polymers generically formulated as [Fe(L)_2_(NCS)_2_]@Guest, L being the bis-monodentate pyridine-type bridging ligands tvp (*trans*-(4,4′-vinylenedipyridine)),^34^ azpy (4,4′-azopyridine),^35^ bpbd (2,3-bis(4′-pyridyl)-2,3-butanediol),^36,37^ bpe (1,2-bis(4′-pyridyl)ethane),^38^ and bped (*dl*-1,2-bis(4′-pyridyl)-1,2-ethanediol)^39^ has had a pivotal influence on the field. Recently, a number of clathrates of the type [Fe(tvp)_2_(NCS)_2_]@Guest (**1@G**) were reported with a variety of guests such as water, acetonitrile, dimethylacetamide, dimethylsulfoxide, benzonitrile, benzaldehyde, and nitrobenzene.^40^ From this study, we realized that the **1@4(CH**_**3**_**CN·H**_**2**_**O)** clathrate afforded large, robust, and rather stable single crystals in relatively good yield. Thus, we decided to investigate this material as a precursor for systematic syntheses of new clathrate homologues via a single-crystal to single-crystal (SCSC) procedure. For this study, we selected a series of halogeno-benzene derivatives (PhX_*n*_: X = Cl (*n* = 1, 2); X = F (*n* = 1–3, 4 (two isomers), 5, 6); X = Br (*n* = 1)) as suitable guests, since they are liquids at room temperature. Thus, here we report the synthesis, via an SCSC procedure, the structures and physical characterizations of the corresponding clathrates generically formulated as **1@*x*PhX**_***n***_. The structural, magnetic, and calorimetric data confirm that all of the clathrates show complete LS ↔ HS transitions. The clathrate **1@2PhCl**_**2**_ undergoes an uncommon four-step SCO behavior that seems to be correlated with the different orientation of the aromatic guests docked in the channels.

## Results

### Synthesis and Thermal Stability

The starting single crystals of the precursor **1@4(CH**_**3**_**CN·H**_**2**_**O)** were prepared by liquid–liquid diffusion in test tubes.^40^ The corresponding species **1@2PhX**_***n***_ were prepared by soaking single crystals of **1@4(CH**_**3**_**CN·H**_**2**_**O)** in pure liquids of **PhX** = **PhF**, **Ph(1,2)-F**_**2**_, **Ph(1,2,3)-F**_**3**_**, Ph(1,2,3,4)-F**_**4**_, **Ph(1,2,4,5)-F**_**4**_, **PhF**_**5**_, **PhF**_**6**_, **PhCl**, **Ph(1,2)-Cl**_**2**_, and **PhBr** for the required time to replace the CH_3_CN and H_2_O molecules from the channels of **1**. Thermogravimetric analyses (TGA) of freshly prepared single crystals reveal that all compounds decompose in three well-defined steps (Figure S1). A first step, involving the loss of two molecules of PhX_*n*_, takes place in the interval 300–450 K, being centered at ca. 380–448 K depending on the derivative. Then a second step centered at ca. 480–540 K follows, involving a loss of weight consistent with one molecule of tvp, and finally, the third step centered at ca. 605–650 K involves the loss of the second tvp ligand and two SCN groups, the remaining mass being reasonably consistent with a residue of FeO at around 800 K (Figure S1). All of the guests are stable inside the pores except for the lightest ones, i.e. PhCl, PhF_*x*_ (*x* = 1, 2 and 3), and PhBr, which are loosely attached to the walls of the channels and gradually desorb with time (see also the single-crystal section). For example, **1@2PhF** loses ca. 19% of its weight in 2 days, consistent with the desorption of 1.4 molecules of PhF. This effect markedly decreases with an increase in the molecular weight of the guest: e.g., the losses of weight for the homologous PhCl and PhBr clathrates in the same period are ca. 6.2% (∼0.45 molecule) and 5% (∼0.3 molecule), respectively (see Figure S2). However, elemental analyses of freshly prepared samples of these derivatives support the presence of two guest PhX_*n*_ molecules, similarly to the stable members of the series, a fact also confirmed by the crystal structure of the PhCl_2_ and PhF_*n*_ (*n* = 4–6) clathrates (see the next sections).

### Spin-Crossover Behavior

**Figure 1:**
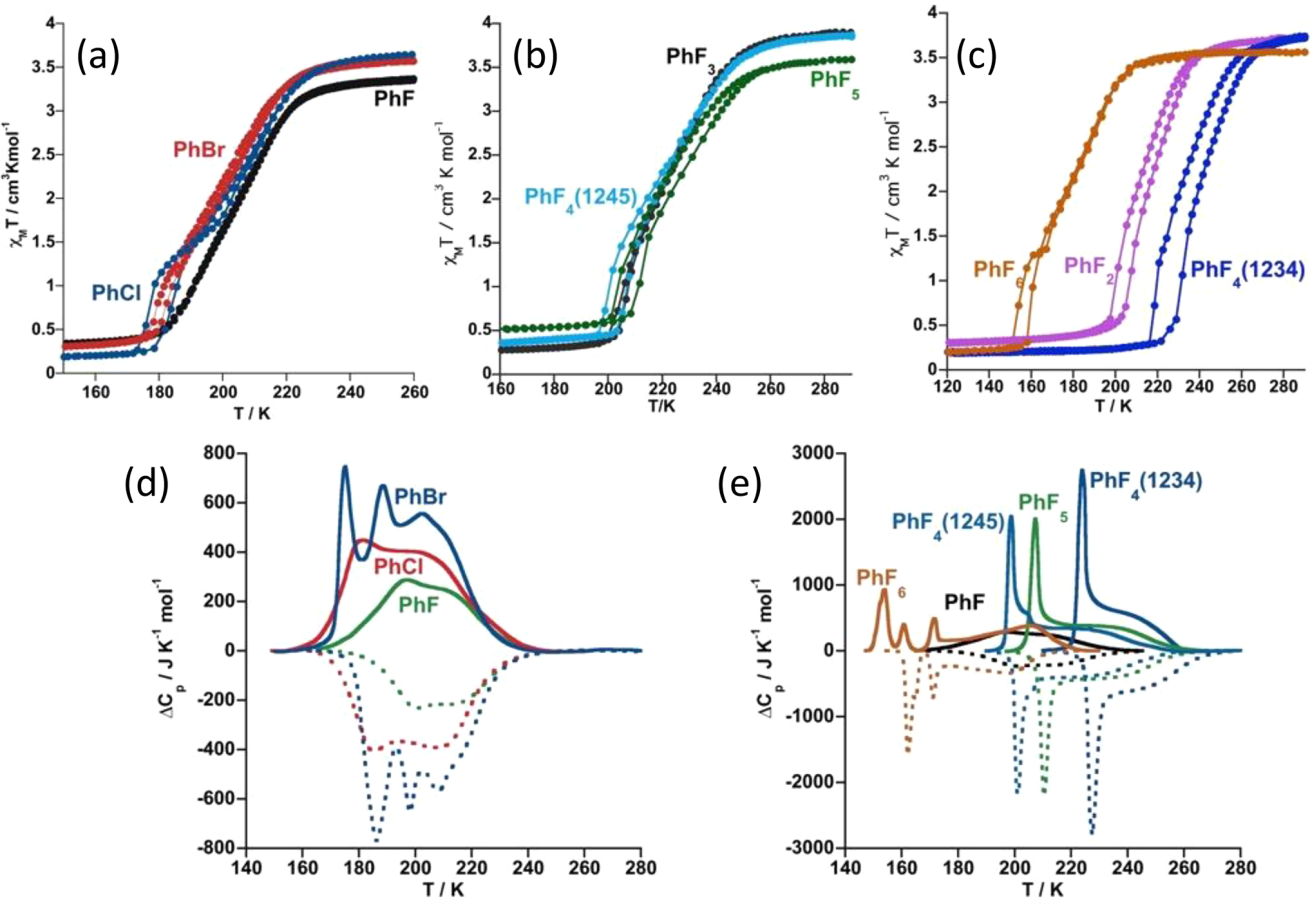
Thermal dependence of χ_M_*T* (a–c) and Δ*C*_p_ (d, e) for **1@2PhX**_**n**_. Solid and dotted lines in (d) and (e) correspond to the cooling and heating modes, respectively.

**Figure 2:**
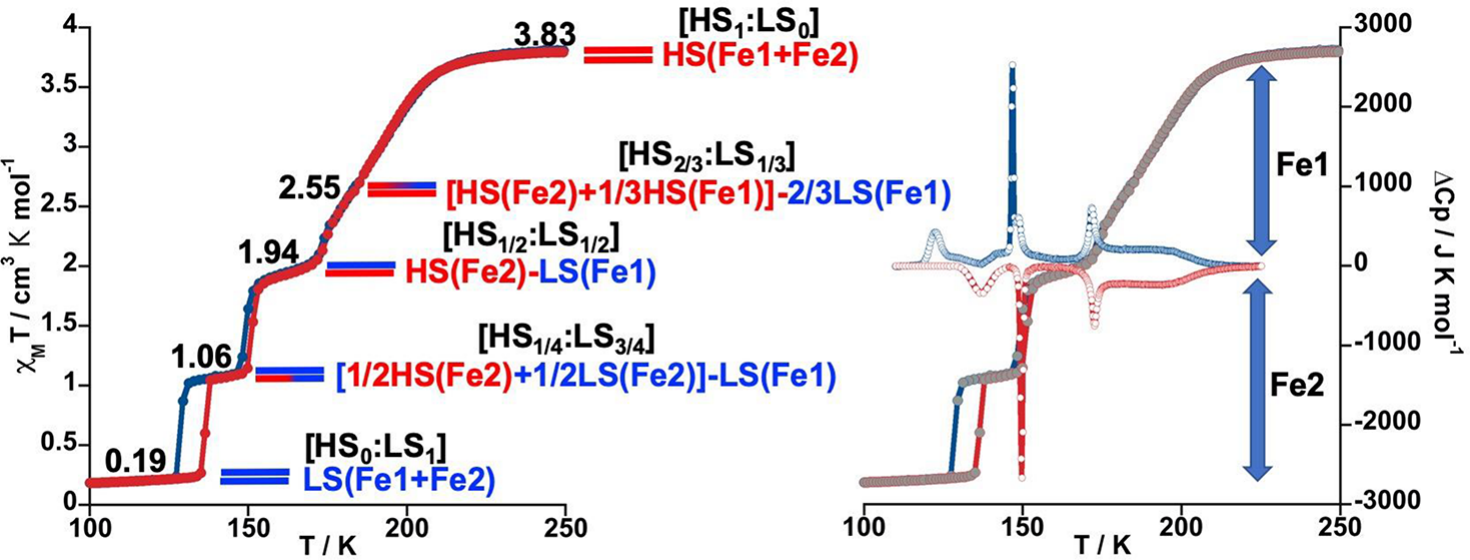
SCO properties of **1@2PhCl**_**2**_. (left) Thermal dependence of χ_M_*T*. Horizontal bars represent the evolution of the spin state (LS blue/HS red) for the two crystallographically distinct Fe1 and Fe2 centers. (right) Overlay of the thermal dependence of Δ*C*_p_ and χ_M_*T* plots.

The SCO properties of freshly prepared crystals of **1@2PhX**_***n***_ were analyzed from the thermal dependence of the χ_M_*T* product and Δ*C*_p_, where χ_M_ is the molar magnetic susceptibility monitored using a SQUID susceptometer (*T* scan rates 1–2 K/min), *T* is the temperature, and Δ*C*_p_ represents the variation of the anomalous specific heat capacity derived from differential scanning calorimetry (*T* scan rates 5–10 K/min) (see the Experimental Section). χ_M_*T* and Δ*C*_p_ vs *T* plots are displayed in Figures 1 and 2 (DSC vs *T* plots for **1@2PhF**_***n***_, *n* = 2, 3, are shown in Figure S3).

**Figure 3:**
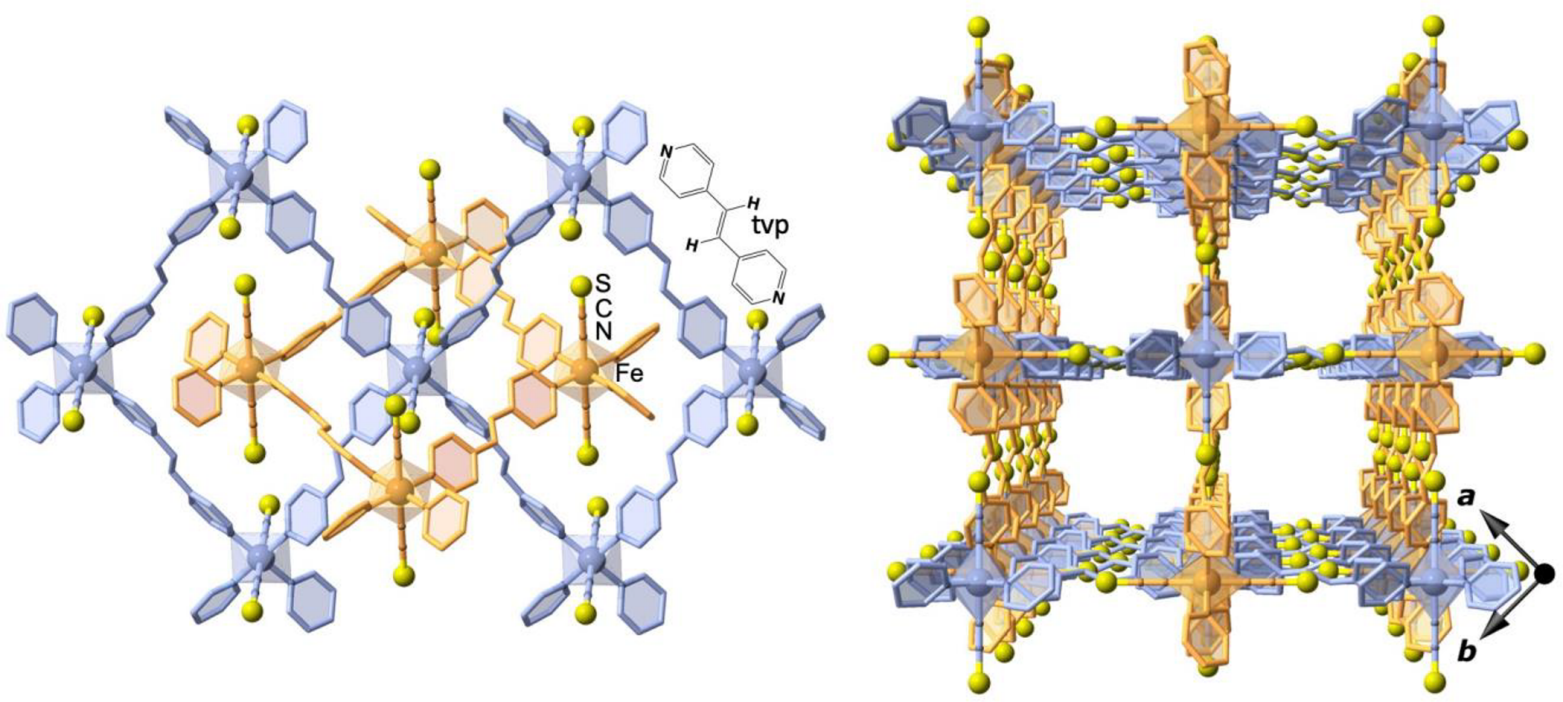
(left) View of two orthogonally interpenetrated [Fe(tvp)_2_(NCS)_2_]_*n*_ layers emphasizing the pseudo-octahedral coordination environment of the Fe^II^ nodes and their relative orientation. (right) Perspective view down the *c* direction of the stacking of the two different sets of layers defining wide square-shaped channels. Yellow spheres represent the S atoms of the SCN^–^ groups. The two independent orthogonally interlocked stacks of layers are shown in orange and blue.

At room temperature, the χ_M_*T* product (3.39–3.86 cm^3^ K mol^–1^) is in the range typically observed for an Fe^II^ center in the HS state. Upon cooling, χ_M_*T* remains almost constant above a certain interval of temperatures but then decreases, first gradually at intermediate temperatures and then more abruptly at lower temperatures, reaching values in the range 0.08–0.25 cm^3^ K mol^–1^, which involves HS molar fractions between ca. 0% and 6%, indicating that the SCO is practically complete (Figure 1a–c). The clathrate **1@2PhCl**_**2**_ displays a four-step SCO associated with the presence of two crystallographically distinct Fe^II^ centers (Fe1 and Fe2; see structure) and average characteristic temperatures defined by the maxima described by ∂(χ_M_*T*)/∂*T* centered at ca. *T*_c_ = 192 K (first), 173 K (second), 150 K (third) and 132 K (fourth), which correspond reasonably well to the switches between the states [HS_1_:LS_0_] ↔ [HS_2/3_:LS_1/3_] ↔ [H*S*_1/2_:L*S*_1/2_] ↔ [HS_1/4_:LS_3/4_] ↔ [HS_0_:LS_1_] defined by the χ_M_*T* intervals 3.83 ↔ 2.55 ↔ 1.94 ↔ 1.06 ↔ 0.19 cm^3^ K mol^–1^, respectively (Figure 3). Except for the PhF derivative, the χ_M_*T* vs *T* plot in the heating mode does not match that of the cooling mode, particularly in the abrupt part of the SCO at low temperatures, thereby defining a narrow asymmetric thermal hysteresis in the interval range 2–10 K. The average equilibrium temperature, *T*_1/2_^av^, at which the molar fractions of the HS and LS states, γ_HS_ and γ_LS_, are equal to 0.5 (where the variation of the free energy Δ*G* = 0) is shown in Table 1 for all derivatives. These *T*_1/2_^av^ values have been calculated by taking into account that (χ_M_*T*)_1/2_ = {[(χ_M_*T*)_HT_ − (χ_M_*T*)_LT_]/2} + (χ_M_*T*)_LT_, (χ_M_*T*)_HT_ and (χ_M_*T*)_LT_ being the χ_M_*T* values at high (ca. 280–300 K) and low- (ca. 100 K) temperatures, where the compounds are practically HS and LS, respectively.

**Table 1:** Thermodynamic Parameters for **1@2PhX**_***n***_

clathrate	*T*_1/2_^av^/K	Δ*H*^av^/kJ mol^–1^	Δ*S*^av^/J K^–1^ mol^–1^
**1@2PhCl**	198	19.01	96.0
**1@2PhCl**_**2**_	162	16.86	104.1
**1@2PhF**	203	9.67	47.7
**1@2PhF**_**2**_	213	18.62	87.4
**1@2PhF**_**3**_	220	21.91	99.6
**1@2PhF**_**4**_**(1,2,3,4)**	235	26.63	113.3
**1@2PhF**_**4**_**(1,2,4,5)**	217	21.98	101.3
**1@2PhF**_**5**_	220	22.78	103.5
**1@2PhF**_**6**_	175	17.21	98.4
**1@2PhBr**	200	24.0	120.0

The thermal dependence of Δ*C*_p_, in the cooling and heating modes, reflects reasonably well the SCO behavior described by the magnetic measurements. The wide shoulder dominating the high-temperature range corresponds to the more gradual part of the SCO (smaller slope), while the sharp maximum at lower temperatures reflects the increase of slope in the χ_M_*T* vs *T* curve. The four steps described for the PhCl_2_ derivative from the χ_M_*T* vs *T* plot are quite well reproduced by the Δ*C*_p_ vs *T* plot. For each compound, the average variations of enthalpy, Δ*H*^av^, obtained from integration of the area below the Δ*C*_p_ vs *T* curves and the corresponding average variations of entropy calculated as Δ*S*^av^ = Δ*H*^av^/*T*_1/2_^av^ are gathered in Table 1. These enthalpy and entropy values are in general larger than those usually observed for Fe^II^ SCO compounds but are consistent with those observed for previously reported clathrates of the porous framework [Fe(tvp)_2_(NCS)_2_]_*n*_.^40^

### Structure of **1@*x*PhX_*n*_**

Single-crystal analyses were carried out for all the clathrates under study using freshly prepared samples. The lightest guests of the **1@2PhX**_***n***_ series, PhCl and PhF_*n*_ (*n* = 1, 2, 3), were found to be strongly disordered and the diffuse electron density could not be modeled. The scattering contributions of the disordered guests were removed using the solvent mask procedure implemented in Olex2.^41^ The electron density evaluated by following this protocol suggests that the amount of guest in the pores is reduced by ca. 25–40%, presumably favored by the continuous N_2_ jet during the single-crystal X-ray diffraction measurements, giving the estimated content of guest *x* ≈ 1.14–1.32. In contrast, **1@2PhCl**_**2**_ and the more hightly substituted fluorobenzene guests, *n* = 4–6, were perfectly localized in the channels. Despite this, the *R* value for the clathrate **1@2PhF**_**4**_**(1,2,4,5)** (*R*_int_ = 0.13) is surprisingly high, most probably due to fractures in the crystals or twinning. However, the data have sufficient quality to allow an accurate determination of the structure of both the host framework and the guests. Only the cell parameters could be obtained for **1@*x*PhBr** (see Table S1).

Except for **1@2PhCl**_**2**_, all of the structures investigated are centrosymmetric. The PhX_*n*_ derivatives with X = F, Cl, Br are isostructural and display the tetragonal space group *P*4*/ncc*, while the remaining centrosymmetric clathrates adopt the orthorhombic space group *Pccn*. The noncentrosymmetric PhCl_2_ derivative adopts the space group *P*4̅2_1_*c* in the whole range of temperatures measured and contains two enantiomeric forms. All of the clathrate compounds show the same basic structure for the metal–organic framework,^6,11^ the main difference being the guest molecule and its location in the channels. Crystallographic data for the **1@*x*PhX**_***n***_ series are gathered in Tables S2–S4 while Tables S5–S7 contain a selection of relevant bond lengths and angles.

Let us recall the structure of the centrosymmetric porous host frameworks. It is constituted of a unique type of pseudo-octahedral Fe^II^ site which defines the nodes of the framework. Each Fe^II^ node is equatorially connected to four adjacent equivalent nodes lying in the same plane through four bis-bidentate tvp bridging ligands, while two terminal SCN^–^ groups complete the axial positions of the octahedron. The resulting 2D layered [Fe(tvp)_2_(NCS)_2_]_*n*_ coordination polymers define two independent and orthogonally interpenetrated stacks giving large square-shaped channels that confer a porous nature to the compound (Figure 3).

The structures **1@*x*PhX**_***n***_ with X = Cl (*x* ≈ 1.2, *n* = 1), F (*x* ≈ 1.1–1.3, *n* = 1–3), and F (*x* = 2, *n* = 5) were analyzed at 140/260 K (PhCl), 130/260 K (PhF), 140/263 K (PhF_2_), and 120/260 K (PhF_3_ and PhF_5_), where the compounds are in the LS/HS state. In the HS state the average Fe–N bond length ⟨Fe–N⟩ _av_ is serially 2.170(4), 2.171(4), 2.158(3), 2.170(3) and 2.156(7) Å and shortens 0.194, 0.200, 0.181, 0.199, and 0.189 Å down to 1.976(3), 1.971(4), 1.977(2), 1.971(3), and 1.967(4) Å in the LS state. The apparent partial loss of the guest in four of the five clathrates does not significantly affect the variations of ⟨Fe–N⟩_av_, since they are perfectly consistent with what is expected for a complete HS ↔ LS conversion, in agreement with the magnetic behavior. In this respect, it is worth pointing out again that complete loss of the guest induces stabilization of the HS state for the empty framework.^40^ Then, below a certain threshold value of *x* for the aforementioned derivatives it is expected that the incompleteness of the SCO will grow more and more as *x* approaches zero. For the derivatives **1@2PhF**_***n***_ with *n* = 4 (1,2,3,4- and 1,2,4,5-isomers) and *n* = 6 the structures were measured at 120 K, giving respectively ⟨Fe–N⟩_av_ bond lengths of 1.974(2), 1.971(6) and 1.991(8) Å, typical of the LS state.

**Figure 4:**
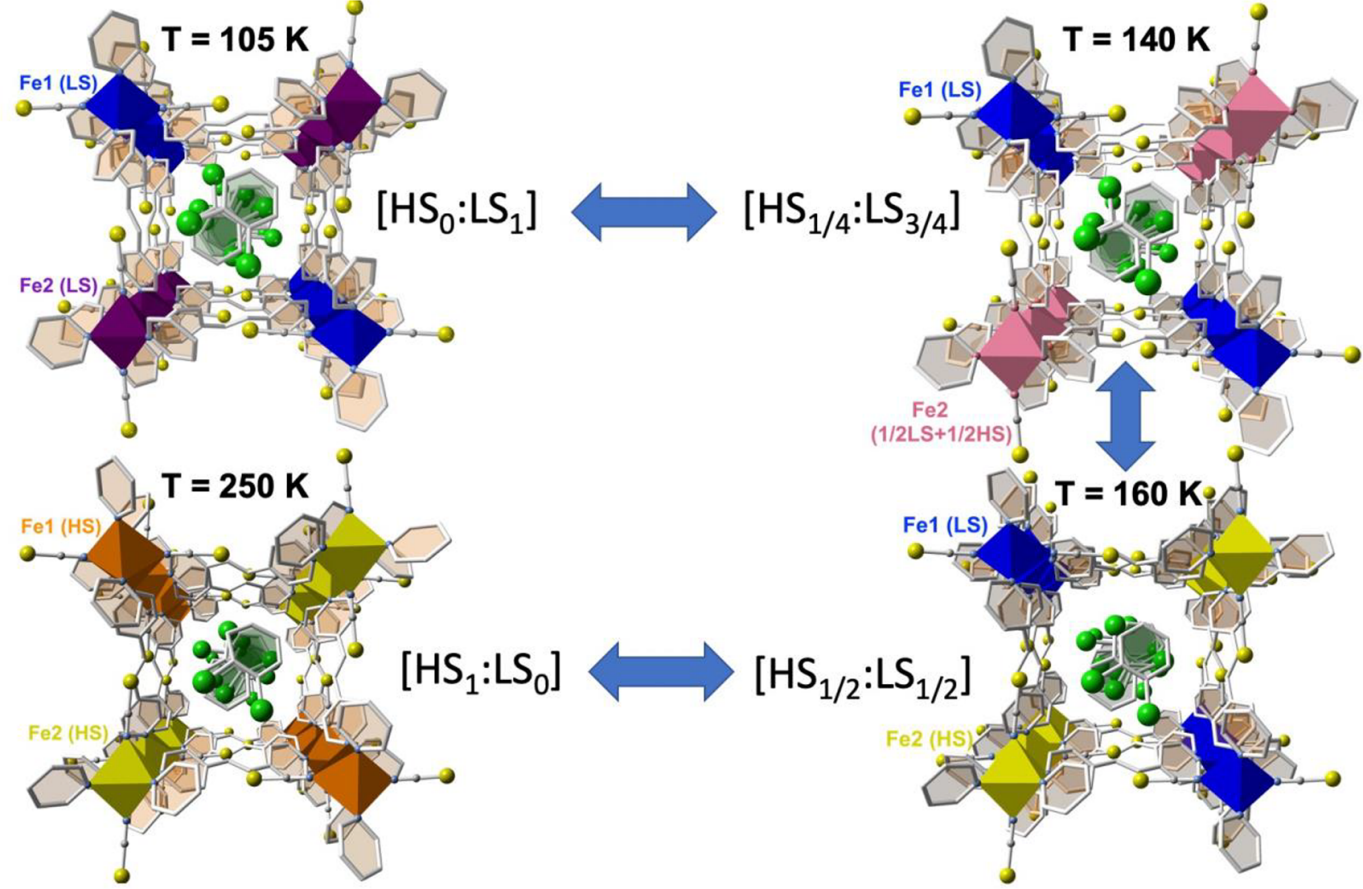
Fragment of the structure of **1@2PhCl**_**2**_ showing the guest PhCl_2_ molecules in the middle of the channels and the spin-state change of the Fe^II^ octahedral nodes. Color codes: Fe1 (LS), blue; Fe2 (LS), garnet; Fe2 (HS), yellow; Fe1 (HS), brown; Fe2 (1/2HS+1/2LS), pink.

Because of the multistep SCO nature of **1@2PhCl**_**2**_, its structure was analyzed at four characteristic temperatures (105, 140, 160, and 250 K), which correspond to the well-defined plateaus between the successive steps. The poorly marked plateau and its pronounced slope prevented us from analyzing it at around 194 K, which corresponds to a ca. HS_2/3_:LS_1/3_ state (see Figure 2, left). The structure of the host framework is essentially the same as for the rest of the **1@*x*PhX**_***n***_ family, but in contrast, there are two crystallographically distinct Fe^II^ pseudo-octahedral centers (Fe1 and Fe2) (Table S5) featuring practically the same ⟨Fe–N⟩_av_ values, 1.980(4) Å (Fe1) and 1.983(4) Å (Fe2), at 105 K. The only remarkable difference is observed for the angular distortion parameters ∑(Fe_i_) (sum of the deviation from 90° of the 12 cis angles of the [FeN_6_] octahedron), which are 10.68 and 7.45° for Fe1 and Fe2, respectively. Upon heating, the Fe1 site remains within the typical limits of the LS state (⟨Fe1–N⟩_av_ = 1.982(3) Å (140 K) and 1.991(3) Å (160 K)), while for the Fe2 site ⟨Fe2–N⟩_av_ substantially increases by 0.092 Å (140 K) and 0.084 Å (160 K) up to 2.074(3) and 2.158(3) Å, respectively. The ∑ parameter slightly increases for Fe1 (to 11.21 and 11.83°) and increases more markedly for Fe2 (to 8.85 and 10.57°) upon heating to 140 and 160 K, respectively. At 250 K, the ⟨Fe1–N⟩ _av_ value increases by 0.185 Å up to 2.176(5) Å, while an additional increase of 0.023 Å is observed for ⟨Fe2–N⟩_av_ to reach a final value of 2.181(5) Å. At 250 K, ∑ values are 13.92 and 12.07° for Fe1 and Fe2, respectively. The global ⟨Fe(1+2)–N⟩_av_ average values move following the sequence 1.981(4) Å (105 K), 2.028(3) Å (140 K), 2.074(3) Å (160 K), and 2.178(5) Å (250 K). These results indicate that the differences in ⟨Fe–N⟩_av_ between the HS and LS states, 0.196 Å (Fe1), 0.198 Å (Fe2), and 0.197 Å (Fe1 + Fe2), are consistent with the occurrence of complete SCO and, the sequence of changes in ⟨Fe–N⟩_av_, ⟨Fe1–N⟩_av_, and ⟨Fe2–N⟩_av_ agree reasonably with the sequence deduced from the χ_M_*T* vs *T* plot for **1@2PhCl**_**2**_. Although from the structural data no ordering was observed for the distribution of the LS and HS Fe2 centers at 140 K, it is reasonable to suggest that they alternate parallel to *c*, as shown in Figure 4 (pink octahedra).

The clathrates exhibiting low- or medium-disordered guest molecules, i.e. **1@2PhF**_***n***_ with *n* = 4 (1,2,3,4- and 1,2,4,5-substituted), 5, 6 and **1@2PhCl**_**2**_, clearly show the guest aromatic rings filling the nanochannels organized in a more or less displaced face to face fashion held together through π–π interactions. In the LS state, the centroid to centroid separations are respectively 3.568 and 3.417, 3.533, and 3.493 Å for the tetra (1,2,3,4 and 1,2,4,5)- penta-, and hexasubstituted **1@2PhF**_**n**_ derivatives and 3.575 Å for **1@2PhCl**_**2**_. This separation increases by 0.158 and 0.089 Å upon switching from the LS to the HS state for **1@2PhF**_**5**_ and **1@2PhCl**_**2**_, respectively.

**Figure 5:**
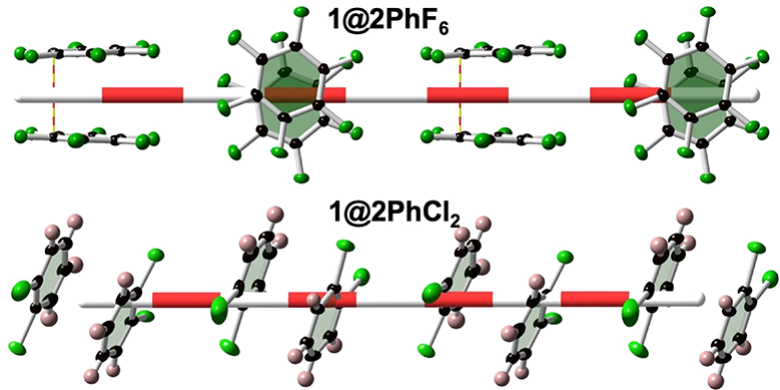
Supramolecular organization of the guests in the nanochannels running along the *z* direction for **1@PhF**_**6**_ and **1@PhCl**_**2**_. The large bicolor bar denotes the center of the pore. The thin bicolor line in **1@PhF**_**6**_ corresponds to the shortest C···C intermolecular distance, 3.239 Å, smaller than the sum of the van der Waals radii (3.4 Å).

It is important to note that the packing of the guest pairs in **1@2PhCl**_**2**_ is radically distinct from that found for the remaining members of the series. Indeed, the PhCl_2_ aromatic rings are practically parallel-oriented to the cross-section surface defined by the nanochannels, while the other PhX_*n*_ rings of the series are perpendicularly oriented to it (see Figure 5 and Figure S4). Consequently, the smallest centroid to centroid separation between two consecutive pairs is shorter for **1@2PhCl**_**2**_ (5.607 Å (LS) and 5.457 Å (HS)), these separations being in the interval 7.861–8.242 Å (LS state) for the fluorine derivatives. Despite this, the effective separation between the **PhF**_***n***_ (*n* = 4–6) rings is determined by the shortest F···F distances between consecutive pairs (3.135 Å (*n* = 6), 3.255 Å (LS)/3.337 Å (HS) (*n* = 5), 3.277 Å (*n* = 4 (1,2,3,4)), and 3.364 Å (*n* = 4 (1,2,4,5))), which are somewhat larger than the sum of the van der Waals radii (2.94 Å).

**Figure 6:**
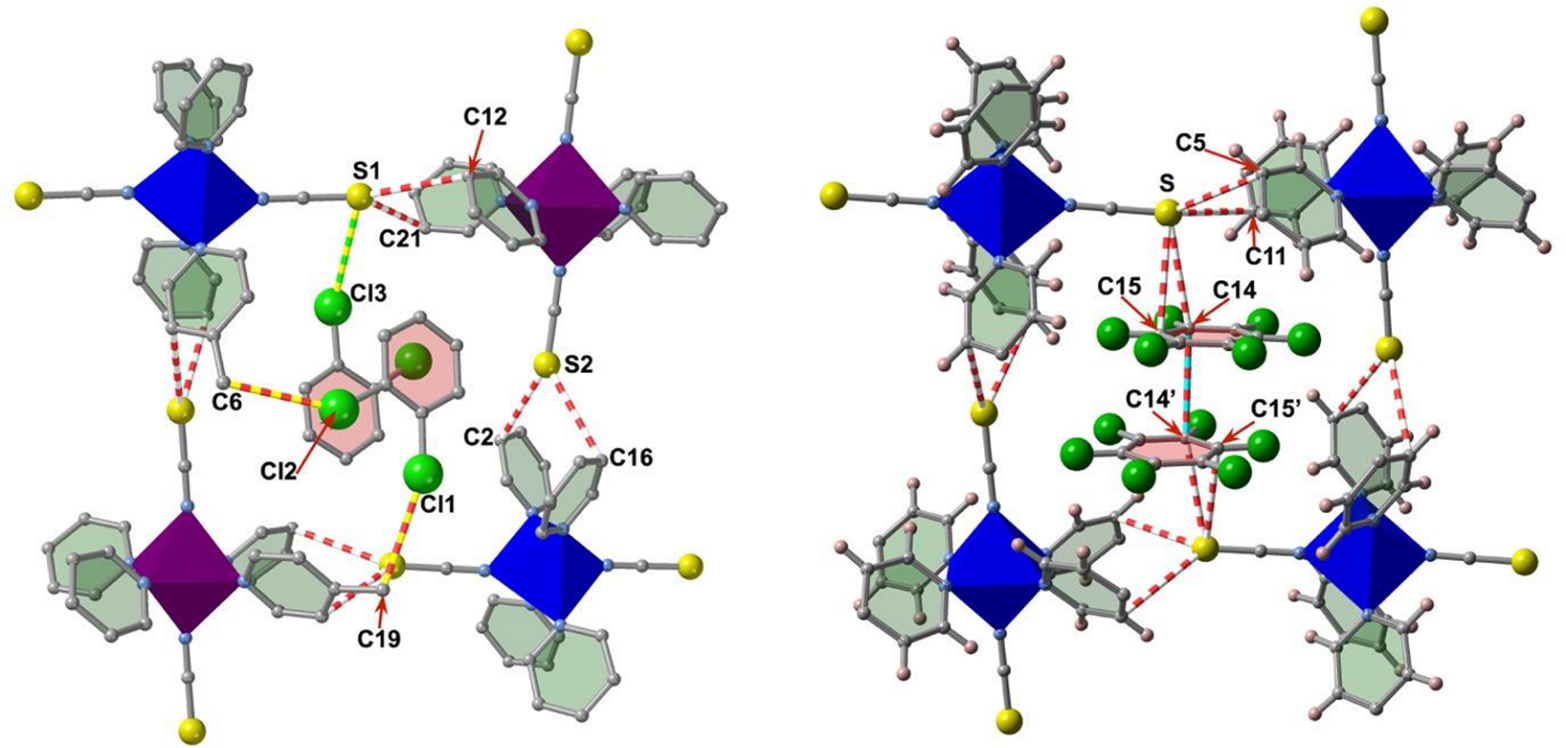
Representative guest–guest (blue–red), host–guest (green–yellow) and host–host (red–white) intermolecular contacts for the LS structures of **1@2PhCl**_**2**_ (104 K) (left) and **1@2PhF**_**6**_ (120 K) (right).

The singular orientation of the guests in **1@2PhCl**_**2**_ determines the nature of the shortest intermolecular host–guest contacts, involving additional Cl···C and Cl···S close distances at 105 K where the two Fe^II^ centers are in the LS state (*d*(Cl1···C19) = 3.410 Å; *d*(Cl2···C6) = 3.380 Å, and *d*(Cl3···S1) = 3.559 Å) (Figure 6, left). In contrast, only the most crowded derivatives of the PhF_*n*_ series, i.e. **1@2PhF**_**6**_ and **1@2PhF**_**5**_, display two and one host–guest S···C contact (S···C14 = 3.401 Å and S···C15 = 3.488 Å at 150 K and S···C15 = 3.485 Å, 120 K, respectively) (Figure 6, right, and Figure S5); for the rest of the members these contacts are larger than the sum of the van der Waals radii. A common feature of all the members of the [Fe(tvp)_2_(NCS)_2_@Guest] family is the occurrence of two characteristic host–host C···S interactions in the LS state between the S atom of the NCS^–^ groups of one independent stack of layers and two C atoms of two “cis” pyridines belonging to the other independent interpenetrated stack of layers (see Figure 6 and Figure S5). All of these interactions gradually weaken as the HS is populated (Figure S6).

## Discussion and Conclusions

We reinvestigate here the clathration properties of the host system [Fe(tvp)_2_(NCS)_2_]_*n*_ (**1**) made up of two mutually interpenetrated orthogonal stacks of parallel 2D layers, which afforded the first example of porous SCO.^34^ More precisely, the aim of this study was first to demonstrate the reliability of the **1@4(H**_**2**_**O·CH**_**3**_**CN)** derivative as a precursor for the systematic synthesis of halogeno-benzene clathrates by interchanging the H_2_O and CH_3_CN molecules via single-crystal to single-crystal transformations. This has been achieved by soaking the precursor crystals in the liquid of the appropriate guest PhX_*n*_ (X = F, Cl, Br; *n* = 1–6). The second aim was to investigate the SCO properties of the resulting new clathrates.

With regard to the first aspect, the results clearly show that **1@4(H**_**2**_**O·CH**_**3**_**CN)** is an excellent starting material to obtain high-quality single crystals of the selected halogeno-benzene clathrates **1@2PhX**_***n***_ in good yield. Despite this, the lightest PhX_*n*_ members of the series (PhCl and PhF_*n*_ (*n* = 1–3)) are loosely attached to the walls of the channels of the host **1** and tend to escape. Although this circumstance has not prevented the standard characterization of the corresponding clathrate compounds, it has a marked effect on the single-crystal X-ray measurements, this being the cause of the low nominal loading of guest molecules inferred from the single-crystal analysis. In addition, it contributes to an increase in the high disorder observed for the confined guests, a fact that does not allow us to describe neither their orientation in the channels nor the potential host–guest and guest–guest short contacts. A similar situation was previously reported for the PhCHO and PhNO_2_ clathrates.^40^ However, a picture of the supramolecular organization for the PhCHO molecules has been recently obtained from molecular dynamics simulations,^41^ which agrees reasonably well with the distribution in the channels of a homologous PhCN clathrate directly observed from a single-crystal analysis^40^ (see Figure S7) as well as in the clathrates **1@2PhF**_***n***_ (*n* = 4–6), described here, and in the *p*-dichlorobenzene clathrate of the equivalent orthogonally interpenetrated 2D framework [Fe(bpe)_2_(NCS)_2_]_*n*_.^38c^

As their congeners **1@4(H**_**2**_**O·CH**_**3**_**CN)** and **1@2PhCN**, the simplest **1@*x*PhX** (1 ≤ *x* ≤ 2; X = F, Cl, Br) clathrates crystallize in the space group *P*4/*ncc* under ambient conditions, where the HS state is stable. However, while **1@*x*PhX** retains this space group in a switch to the LS state, the first two undergo a crystallographic phase transition, adopting the space group *Pccn* that involves a change in the crossing angle defined by the interpenetrated 2D frameworks from orthogonal to oblique. In contrast, despite the layers remaining orthogonally interpenetrated, the space group *Pccn* is also observed in the LS state for **1@*x*PhF**_***n***_ (*n* = 2–6). Whatever the space group, the precise supramolecular organizations adopted by the distinct halogeno-benzene molecules in the host channels do not substantially differ from each other and are slightly modulated by the size and number of substituents in the benzene ring, with the remarkable exception of **1@2PhCl**_**2**_. In this case the benzene rings adopt a parallel disposition with respect to the square cross-section of the channels, in contrast to the other members of the series (including **1@2PhCN** and **1@2PhCHO**), which adopt a perpendicular disposition.

The halobenzene clathrates here reported display complete SCO behavior with more or less marked differences both in the characteristic *T*_1/2_ temperatures and in the thermal evolution of the HS/LS population, somewhat modulated by the nature and disposition of the guest in the channels. With regard to the freshly prepared single halogenated benzenes **1@2PhX** (X = F, Cl, Br), the change from F to Br does not significantly influence the *T*_1/2_ value, lying in the range 198–203 K (see Table 1) but promotes simultaneously an increase in cooperativity and the emergence of hysteresis as well as an incipient new SCO step at low temperatures. The hysteresis and the step are not present for the F derivative but are apparent and become more noticeable on moving from Cl to Br (see Figure S8). Both features are also obvious for the **1@2PhF**_***n***_ (n = 2–6) derivatives. Furthermore, except for **1@2PhF**_**6**_ characterized by *T*_1/2_ = 175 K, the rest of the series display somewhat larger *T*_1/2_ values (range 213–235 K) in comparison to those observed for the single halogenated benzenes. All of these observations derived from the χ_M_*T* vs *T* plots are reasonably well supported by the DSC measurements, which confirm the occurrence at least two incipient steps.

Although it was much less marked in comparison with the title compounds, the propensity of SCO to occur in two steps with different cooperativities was already noted previously for the clathrates 4(H_2_O·CH_3_CN), 2PhNO_2_, and 2PhCHO. This was associated with the accommodation of the framework and guests to the changes in bond lengths and bond angles of the [Fe^II^N_6_] core produced during the thermally induced SCO. As a consequence, the underlying structural constraints generate intermediate phases that evolve with the degree of HS ↔ LS transformation, a fact that is consistent with the characteristic high Δ*H* and Δ*S* values obtained for the majority of the members of the **1@Guest** family. In particular, up to three structural phases have been identified for **1@2PhCHO** in a recent detailed study, mentioned above, that combines variable-temperature synchrotron X-ray diffraction measurements (to determine the long-range order and symmetry lowering) and molecular dynamics simulations (to explore the structure of the guest confined in the channels).^42^

Coming back to the *T*_1/2_ values, we observed no clear correlation in the series of **1@2PhF**_***n***_ (*n* = 1–6). Only in the case of **1@2PhF**_**6**_, the fact of showing the lowest *T*_1/2_ value of the series clearly seems to be correlated with additional short host–guest contacts taking place between the S atom of the SCN^–^ groups and the C14 (3.401 Å) and C15 (3.488 Å) atoms of the most voluminous guest PhF_6_ (Figure 6, right). Obviously, these additional contacts condition the capability of the host framework **1** to self-adapt to the dimensions imposed by the LS state, thereby inducing elastic constraints that destabilize the LS state, pushing down *T*_1/2_. Contacts between equivalent atoms are slightly larger for the other members of the series and depend on the exact location of the pairs (PhF_*n*_)_2_ in the channels, which is modulated by the number of F atoms and their distribution in the ring. These subtle differences are the only features that may explain the observed *T*_1/2_ values for the remaining members of the series. Connected with this are the singular properties observed for **1@2PhCl**_**2**_. Its average *T*_1/2_ value is even smaller than that of **1@2PhF**_**6**_, a fact that is a consequence of the special disposition adopted by the PhCl_2_ rings in the channels, partially blocking the breathing mode of the host framework and hence destabilizing the LS state. This is supported by the fact that the separation between the aromatic rings of a pair of π–π interacting guests (PhCl_2_)_2_ shrinks upon the HS ↔ LS transformation ca. 56% less (0.089 Å) than was observed for the opposite docking configuration of the (PhX_*n*_)_2_ pairs adopted by the other members of the series, in particular for **1@2PhF**_**5**_. In this system, the separation between the PhF_5_ rings in the pairs (PhX_5_)_2_ decreases 0.158 Å. As a consequence, **1@2PhCl**_**2**_ undergoes an uncommon four-step SCO behavior, three of which have been structurally characterized. The steps correspond to the energy tolls required to overcome the elastic frustration generated by the shortest intermolecular short contacts between the host framework and the PhCl_2_ guests. It is worth noting that no symmetry breaking has been observed at the temperatures investigated. Indeed, the correlation of spin state and structure for the three structurally characterized steps is compatible with the presence of two different Fe^II^ sites, each one residing separately in one of the two orthogonally interpenetrated stacks of 2D layers. During the switching from the [LS_0_–HS_1_] to the [LS_1/2_–HS_1/2_] state, only the stack containing the Fe1 site adopts the LS, while the subsequent steps to the [LS_3/4_–LS_1/4_] and [LS_1_–HS_0_] states each involve half of the Fe2 centers. Despite the well-defined plateau generated by the [LS_3/4_–HS_1/4_] state, no Fe2(LS)–Fe2(HS) order is observed, only an average value between both Fe2 sites. Most likely, this order would require a change in space group that is not observed. Although we have not analyzed the structure of the poorly defined step consistent with the [HS_2/3_–LS_1/3_] state at around 192 K, surely it is subjected to similar constraints. The occurrence of multistepped SCO behavior has recently attracted much attention not only for fundamental reasons, since it provides the possibility to find correlations between different competing elastic interactions in the crystal and the stabilization of ordered [LS_*i*_–HS_*j*_] spin states, but also for practical reasons, since these states could be used to design more advanced complex memories. So far, the number of SCO complexes exhibiting well-documented four-step SCO behavior is small. Indeed, to the best of our knowledge, the examples are limited to nine and all of them are coordination polymers.^14,16−22^ From these examples, it can be inferred that in all of them the stabilization of the intermediate states/steps depends on the presence of subtle elastic interactions stemming from supramolecular short contacts that in turn critically depend on the specific composition of the sample. The difficult control of the intermolecular interactions makes the multistepped SCO behavior a fortuitous event and only the systematic search for suitable new SCO compounds will afford the control of these interactions, the rational design of the SCO-steps and possible applications in the future.

## Experimental Section

### Materials and Reagents

*trans*-(4,4′-Vinylenedipyridine), iron(II) sulfate heptahydrate, potassium thiocyanate, and all of the halobenzenes employed here were obtained from commercial sources and used as received without further purification.

### Synthesis of **1@4(MeCN·H**_**2**_**O)**

This compound was synthesized following a literature procedure.^40^ A slow liquid–liquid diffusion technique (layering) using standard test tubes was used to obtain samples of the precursor **1@4(MeCN·H**_**2**_**O)** constituted essentially of single crystals. The bottom layer contains a mixture of FeSO_4_·7H_2_O (75 mg, 0.27 mmol) and KNCS (52.3 mg, 0.54 mmol) in 5 mL of water, the middle layer is a 1/1 H_2_O/MeCN mixture (10 mL), and the top layer contains a MeCN solution of tvp (98.3 mg, 0.54 mmol) (5 mL). All manipulations were performed under an Ar atmosphere, and the solutions contained very small quantities of ascorbic acid to prevent oxidation of the Fe^II^ ion. Anal. Calcd for C_34_H_40_N_10_O_4_S_2_Fe: C, 52.86; H, 5.18; N, 18.14. Found: C, 53.04; H, 5.11; N, 17.95.

### Synthesis of **1@2PhX**_***n***_

The halobenzene clathrates were synthesized by soaking single crystals of **1@4(CH**_**3**_**CN·H**_**2**_**O)** (ca. 50 mg) in ca. 3 mL of PhX_*n*_ (PhF, Ph(1,2)-F_2_, Ph(1,2,3)-F_3_, Ph(1,2,3,4)-F_4_, Ph(1,2,4,5)-F_4_, PhF_5_, PhF_6_, PhCl, Ph(1,2)-Cl_2_, and PhBr) for ca. 10 days. It is important to note that, depending on the relative density of the guest with respect to that of the precursor crystals, the procedure was slightly modified. For PhX_*n*_ guests less dense than the crystals, the solid–liquid mixture was kept, usually, in small closed vials. However, in the opposite case, to keep the crystals submerged in the liquid it was necessary to use a syringe as a vial closed with a rubber stopper, as shown in Figure S9. The liquid of the mixtures was replaced every day to accelerate the exchange of guests. Freshly prepared samples of **1@2PhX**_***n***_ were used for elemental analyses. Anal. Calcd for C_38_H_30_F_2_N_6_FeS_2_ (**1@2PhF**): C, 62.64; H, 4.15; N, 11.53. Found: C, 51.07; H, 3.92; N, 11.07). Calcd for C_38_H_28_F_4_N_6_FeS_2_ (**1@2PhF**_**2**_): C, 59.69; H, 3.69; N, 10.99. Found: C, 58.83; H, 3.61; N, 10.65. Calcd for C_38_H_26_F_6_N_6_FeS_2_ (**1@2PhF**_**3**_): C, 57.01; H, 3.27; N, 10.50. Found: C, 56.35; H, 3.16; N, 10.28. Calcd for C_38_H_24_F_8_N_6_FeS_2_ (**1@2PhF**_**4**_ [1,2,3,4]): C, 54.56; H, 2.89; N, 10.05. Found: C, 54.11; H, 2.82; N, 9.89. Calcd for C_38_H_24_F_8_N_6_FeS_2_ (**1@2PhF**_**4**_ [1,2,4,5]): C, 54.56; H, 2.89; N, 10.05. Found: C, 54.08; H, 2.75; N, 9.94. Calcd for C_38_H_22_F_10_N_6_FeS_2_ (**1@2PhF**_**5**_): C, 52.31; H, 2.54; N, 9.63. Found: C, 51.89; H, 2.49; N, 9.56. Calcd for C_38_H_20_F_12_N_6_FeS_2_ (**1@2PhF**_**6**_): C, 50.23; H, 2.22; N, 9.25. Found: C, 49.87; H, 2.19; N, 9.17. Calcd for C_38_H_30_Cl_2_N_6_FeS_2_ (**1@2PhCl**): C, 59.93; H, 3.97; N, 11.04. Found: C, 58.41; H, 3.66; N, 10.53. Calcd for C_38_H_28_Cl_4_N_6_FeS_2_ (**1@2PhCl**_**2**_): C, 54.96; H, 3.56; N, 9.88. Found: C, 58.41; H, 3.66; N, 10.53. Calcd for C_38_H_30_Br_2_N_6_FeS_2_ (**1@2PhCl**): C, 53.67; H, 3.56; N, 9.88. Found: C, 52.90%; H, 3.49; N, 9.72.

### Physical Measurements

#### Magnetic Measurements

Variable temperature magnetic susceptibility data were recorded with a Quantum Design MPMS2 SQUID magnetometer equipped with a 7 T magnet, operating at 1 T and at temperatures of 1.8–400 K. Experimental susceptibilities were corrected for diamagnetism of the constituent atoms by the use of Pascal’s constants.

#### Calorimetric and Thermogravimetric Measurements

Differential scanning calorimetry measurements were performed using a Mettler Toledo Model DSC 821e calorimeter. Low temperatures were obtained with an aluminum block attached to the sample holder, refrigerated with a flow of liquid nitrogen, and stabilized at a temperature of 110 K. The sample holder was kept in a drybox under a flow of dry nitrogen gas to avoid water condensation. The measurements were performed using ∼15 mg of microcrystalline samples of **1@2PhX**_***n***_ sealed in aluminum pans with a mechanical crimp. Temperature and heat flow calibrations were made with standard samples of indium by using its melting transition (429.6 K, 28.45 J g^–1^). An overall accuracy of ±0.2 K in temperature and ±2% in the heat capacity is estimated. The uncertainty increases for the determination of the anomalous enthalpy and entropy due to the subtraction of an unknown baseline.

Thermogravimetric analysis was performed on a Mettler Toledo TGA/SDTA 851e instrument, in the 290–1200 K temperature range under a nitrogen atmosphere at a rate of 10 K min^–1^.

#### Single-Crystal X-ray Diffraction

Single-crystal X-ray data were collected on an Oxford Diffraction Supernova diffractometer using graphite-monochromated Mo Kα radiation (λ = 0.71073 Å). A multiscan absorption correction was performed. The structures were solved by direct methods using SHELXS-2014 and refined by full matrix least-squares on *F*^2^ using SHELXL-2014.^43^ Non-hydrogen atoms were refined anisotropically, and hydrogen atoms were placed in calculated positions, refined using idealized geometries (riding model), and assigned fixed isotropic displacement parameters. The following CCDC file numbers contain supplementary crystallographic data for this article. **1@1.2PhCl**: CCDC 2130154 (140 K) and CCDC 2130155 (260 K). **1@2PhCl**_**2**_: CCDC 2130159 (105 K), CCDC 2130158 (140 K), CCDC 2130166 (160 K), and CCDC 2130165 (250 K). **1@1.3PhF**: CCDC 2130162 (130 K) and CCDC 2130157 (260 K). **1@1.14PhF**_**2**_: CCDC 2130156 (140 K). **1@1.2PhF**_**3**_: CCDC 2130163 (150 K). **1@2PhF**_**4**_**(1,2,3,4)**: CCDC 2130160 (150 K). **1@2PhF**_**4**_**(1,2,4,5)**: CCDC 2130161 (150 K). **1@2PhF**_**5**_: CCDC 2130168 (120 K) and CCDC 2130164 (260 K). **1@2PhF**_**6**_: CCDC 2130167 (120 K).

## Supporting Information Available

Thermogravimetric analyses, differential scanning calorimetry, details of the crystal data collection and refinement parameters as well as selected bond distances and angles, supramolecular organization and supramolecular short contacts, perspective view of the structure of **1@2PhCN**, thermal dependence of χ_M_*T* for **1@2PhX** (X = F, Cl, Br), and experimental setup for PhX_*n*_ guests denser than **1@4(CH**_**3**_**CN·H**_**2**_**O)** (PDF)

## Accession Codes

CCDC 2130154–2130168 contain the supplementary crystallographic data for this paper. These data can be obtained free of charge via www.ccdc.cam.ac.uk/data_request/cif, or by emailing data_request@ccdc.cam.ac.uk, or by contacting The Cambridge Crystallographic Data Centre, 12 Union Road, Cambridge CB2 1EZ, UK; fax: +44 1223 336033.
