# Supplementary material for: Halobenzene Clathrates of the Porous Metal–Organic Spin-Crossover Framework [Fe(tvp)2(NCS)2]n. Stabilization of a Four-Step Transition
Source: Inorg Chem. 2022 Mar 2;61(10):4484–93. doi: 10.1021/acs.inorgchem.2c00014 (PMC8924922; doi:10.1021/acs.inorgchem.2c00014)

Supporting Information

Halobenzene clathrates of the porous metal-organic spin-crossover framework $[Fe(tvp)_2(NCS)_2]_n$. Stabilization of a four-step transition.

Alejandro Orellana-Silla,[a] Francisco Javier Valverde-Muñoz,*[a] M. Carmen Muñoz,[b] Carlos Bartual Murgui,[a] Sacramento Ferrer[c] and José Antonio Real*[a]

[a]Instituto de Ciencia Molecular/Departamento de Química Inorgánica, Universidad de Valencia, 46980 Paterna, Valencia, Spain.

[b]Departamento de Física Aplicada, Universitat Politècnica de València, Valencia 46022, Spain.

[c]Departamento de Química Inorgánica, Universidad de Valencia, 46100 Burjassot, Valencia, Spain.

*E-mail: Francisco.Valverde@uv.es, jose.a.real@uv.es

Figure S1. Thermal analysis for the **1@2PhX$_n$** series. Page 2

Figure S2. Thermogravimetric analysis of **1@xPhX**. Page 3

Figure S3. DSC curves for freshly samples of **1@2PhF$_n$** (n = 2 and 3). Page 3

Table S1. Crystal data for **1@PhBr**. Page 3

Table S2. Crystal data for **1@1.2PhCl** and **1@2PhCl$_2$**. Page 4

Table S3. Crystal data for **1@xPhF$_n$** (n = 1, 2, 3). Page 5

Table S4. Crystal data for **1@2PhF$_n$** (n = 4-6) (PhF_4 = 1,2,3,4-PhF_4). Page 6

Table S5. Selected bond lengths [Å] and angles [°] for **1@1.2PhCl** and **1@2PhCl$_2$**. Page 7

Table S6. Selected bond lengths [Å] and angles [°] for **1@xPhF$_n$** (n = 1, 2, 3). Page 7

Table S7. Selected bond lengths [Å] and angles [°]for **1@2PhF$_n$** (n = 4, 5, 6). Page 7

Figure S4. Supramolecular organization for **1@PhF$_5$** and **1@PhF$_4$(1,2,3,4) and 1@PhF$_4$(1,2,4,5)**. Page 8

Figure S5. Supramolecular interactions for **1@2PhF$_4$(1,2,3,4)** and **1@2PhF$_5$**. Page 8

Figure S6. Thermal variation of relevant supramolecular contacts for **1@2PhCl$_2$**. Page 9

Figure S7. View of a fragment of the structure of **1@2PhCN** (LS, 120 K) down [001]. Page 10

Figure S8. Thermal dependence of $\chi_M T$ for **1@2PhX** (X = F, Cl, Br). Page 10

Figure S9. Experimental set up for PhX_n guests denser than **1@4(CH$_3$CN·H$_2$O)**. Page 11

Figure S1. Thermal analysis for the **1@2PhX$_n$** series.

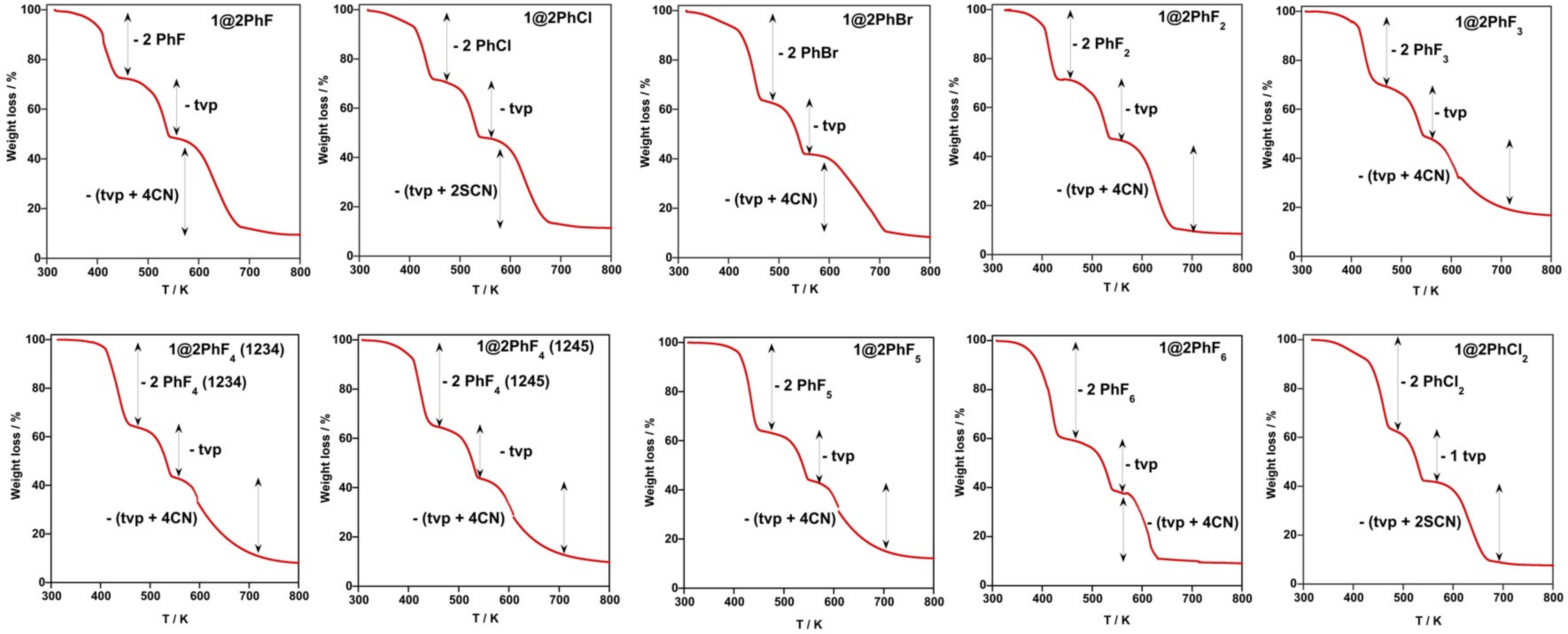

Figure S2. Thermogravimetric analysis of **1@xPhX** [X = F (left), Cl (middle) and Br (right)]. Blue and red lines are the TGAs measured for a freshly prepared sample and after two days elapsed.

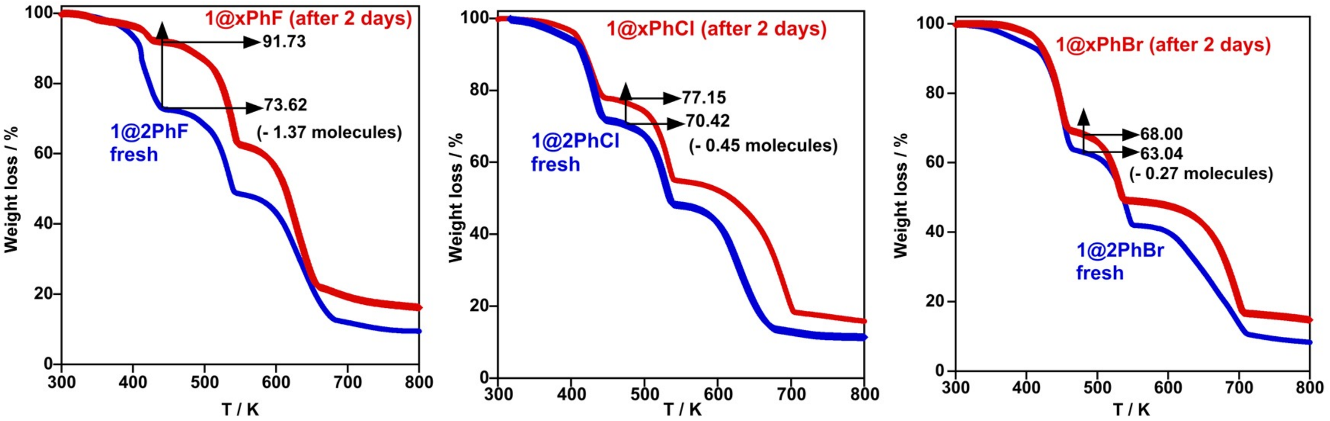

Figure S3. DSC curves for freshly samples of **1@2PhF$_n$** (n = 2 and 3).

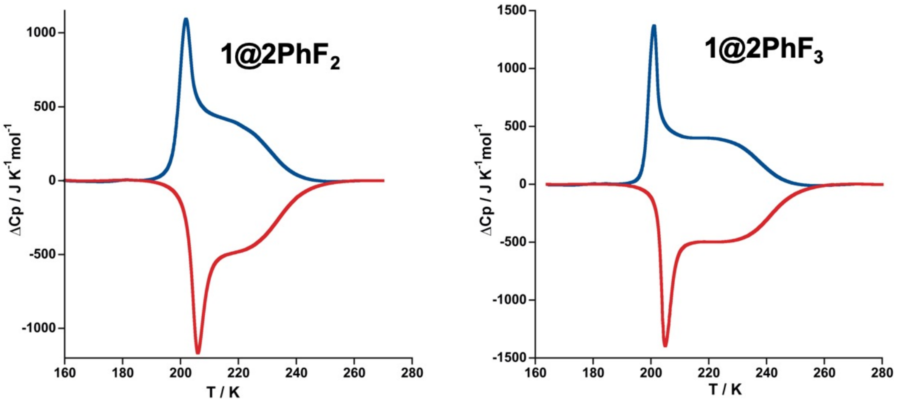

Table S1. Crystal data for **1@PhBr**.

	**1@PhBr**		**1@2PhF$_4$(1245)**
**T/K**	**140**	**255**	**150**
Crystal system	tetragonal		orthorhombic
Space group	P4/ncc		Pccn
a (Å)	15.1053(9)	15.8885(8)	14.7290(7)
b (Å)			15.5930(6)
c (Å)	15.8858(11)	15.6842(13)	15.7660(7)
V (Å^3)	3624.7(5)	3959.4(5)	3621.0(3)

Table S2. Crystal data for **1@1.2PhCl** and **1@2PhCl$_2$**.

	**1@1.2PhCl**		**1@2PhCl$_2$**			
**T / K**	**140 K**	**260 K**	**105 K**	**140 K**	**160 K**	**250 K**
Empirical formula	$C_{33.2}H_{26}N_6S_2Cl_{1.2}Fe$		$C_{38}H_{28}N_6S_2Cl_4Fe$			
*M*r	671.51		830.43			
Crystal system	tetragonal		tetragonal			
Space group	P4/ncc		P-4$2_1$c			
a (Å)	15.1644 (4)	15.8388(4)	21.7385(2)	21.8658(3)	22.0097(2)	22.4856(5)
c (Å)	15.7832(4)	15.7371(7)	15.5582(3)	15.6154(3)	15.6600(2)	15.7327(6)
V (Å^3)	3629.5(2)	3948.0(3)	7352.2(2)	7465.9(3)	7586.1(2)	7954.5(5)
Z	4	4	8			
D_c (mg cm^{-3})	1.229	1.130	1.500	1.478	1.454	1.387
F(000)	1382	1382	3392	3392	3392	3392
μ (Mo-K$_\alpha$) (mm^{-1})	0.648	0.596	0.853	0.840	0.827	0.788
Crystal size (mm)	0.10x0.10x0.18		0.10x0.15x0.15			
No. of total reflections	2515	2423	10012	10189	10346	10780
No. of reflections [$I>2\sigma(I)$]	2073	1299	6953	8837	8661	7072
R [$I>2\sigma(I)$]	0.0637	0.0530	0.0537	0.0446	0.0511	0.0729
wR [$I>2\sigma(I)$]	0.1338	0.1621	0.0962	0.1061	0.1207	0.1809
S	1.055	1.046	1.036	1.016	1.037	1.027

$R_1 = \Sigma\, ||Fo| - |Fc|| \,/\, \Sigma\, |Fo|$; $wR = [\, \Sigma\, [w(Fo^2 - Fc^2)^2] \,/\, \Sigma\, [w(Fo^2)^2]]^{1/2}$.
$w = 1/\, [\boldsymbol{\sigma}^2\, (Fo^2) + (m\, P)^2 + n\, P]$ where $P = (Fo^2 + 2Fc^2) / 3$;
m = 0.0258 (**1**), 0.0838 (**2**), 0.0392 (**3**), 0.0545 (**4**), 0.0579 (**5**) and 0.0946 (**6**);
n = 12.2415 (**1**), 0.0273 (**2**), 2.0362 (**3**), 5.1043 (**4**), 5.8733 (**5**) and 7.0544 (**6**)

Table S3. Crystal data for **1@xPhF$_n$** (n = 1, 2, 3).

	**1@1.3PhF**		**1@1.14PhF$_2$**	**1@1.2PhF$_3$**
Empirical formula	$C_{33.92}H_{26.6}F_{1.32}N_6S_2Fe$		$C_{32.84}H_{24.56}F_{2.28}N_6S_2Fe$	$C_{33.2}H_{23.6}F_{3.6}N_6S_2Fe$
T / K	**130 K**	**260 K**	**140 K**	**150 K**
*M*r	663.30		666.51	694.95
Crystal system	tetragonal	tetragonal	orthorhombic	orthorhombic
Space group	P4/nccc	P4/nccc	Pccn	Pccn
a (Å)	15.1126(2)	15.8431(4)	15.8834(3)	15.1950(8)
b (Å)			15.0986(3)	15.3146(8)
c (Å)	15.8843(3)	15.7488(6)	15.0982(3)	15.6514(6)
V (Å^3)	3627.82(14)	3953.0(3)	3620.82(11)	3642.2(3)
Z	4	4	4	4
D_c (mg cm^{-3})	1.214	1.115	1.223	1.267
F(000)	1368	1368	1368	1421
μ (Mo-K$_\alpha$) (mm^{-1})	0.567	0.521	0.572	0.577
Crystal size (mm)	0.12x0.16x0.16		0.10x0.10x0.18	0.06x0.06x0.18
No. of total reflections	2355	2655	4802	4935
No. of reflections [$I>2\sigma(I)$]	1776	1358	3695	2564
R [$I>2\sigma(I)$]	0.0388	0.0510	0.0571	0.0593
wR [$I>2\sigma(I)$]	0.1039	0.1543	0.1817	0.1742
S	1.063	1.042	1.085	1.073

$R_1 = \Sigma ||Fo| - |Fc|| / \Sigma |Fo|$; $wR = [\Sigma [w(Fo^2 - Fc^2)^2] / \Sigma [w(Fo^2)^2]]^{1/2}$.
$w = 1/ [\sigma^2 (Fo^2) + (m P)^2 + n P]$ where $P = (Fo^2 + 2Fc^2) / 3$;
m = 0.0394 (**1**), 0.0687 (**2**), 0.0807 (**3**) and 0.0784 (**4**)
n = 2.3326 (**1**), 0.4289 (**2**), 6.0896 (**3**) and 1.5531 (**4**)

Table S4. Crystal data for **1@2PhF$_n$** (n = 4-6) (PhF_4 = 1,2,3,4-PhF_4).

	**1@2PhF$_4$**	**1@2PhF$_5$**		**1@2PhF$_6$**
Empirical formula	$C_{38}H_{24}F_8N_6S_2Fe$	$C_{38}H_{22}F_{10}N_6S_2Fe$		$C_{38}H_{20}F_{12}N_6S_2Fe$
T / K	**150 K**	**120 K**	**260 K**	**120 K**
*M*r	836.60	872.58		908.57
Crystal system	orthorhombic	orthorhombic		orthorhombic
Space group	Pccn	Pccn		Pccn
a (Å)	14.7906(4)	14.7775(7)	15.253(2)	14.8734(2)
b (Å)	15.5953(4)	15.7845(10)	16.3554(14)	15.8488(3)
c (Å)	15.7171(6)	15.5340(8)	15.7169(14)	15.4863(2)
V (Å^3)	3625.4(2)	3623.4(3)	3920.9(7)	3650.52(10)
Z	4	4		4
D_c (mg cm^{-3})	1.533	1.600	1.478	1.653
F(000)	1696	1760	1760	1824
μ (Mo-K$_\alpha$) (mm^{-1})	0.611	0.622	0.575	0.629
Crystal size (mm)	0.05x0.05x0.18	0.05x0.05x0.10		0.08x0.08x0.14
No. of total reflections	4882	3642	4031	4839
No. of reflections [$I>2\sigma(I)$]	2736	2292	1620	3811
R [$I>2\sigma(I)$]	0.0511	0.0705	0.1062	0.0370
wR [$I>2\sigma(I)$]	0.0945	0.1856	0.2772	0.0846
S	1.033	1.123	1.004	1.045

$R_1 = \Sigma\,||Fo| - |Fc||\,/\,\Sigma\,|Fo|$; $wR = [\,\Sigma\,[w(Fo^2 - Fc^2)^2]\,/\,\Sigma\,[w(Fo^2)^2]]^{1/2}$.
$w = 1/\,[\boldsymbol{\sigma}^2\,(Fo^2) + (m\,P)^2 + n\,P]$ where $P = (Fo^2 + 2Fc^2)\,/\,3$;
m = 0.0340 (**1**), 0.0868 (**2**), 0.1798 (**3**) and 0.0351 (**4**)
n = 2.2818 (**1**), 4.3228 (**2**), 0.0000 (**3**) and 2.8860 (**4**)

Table S5. Selected bond lengths [Å] and angles [°] for **1@1.2PhCl** and **1@2PhCl$_2$**.

	**1@1.2PhCl**		**1@2PhCl$_2$**			
**T / K**	**140 K**	**260 K**	**105 K**	**140 K**	**160 K**	**250 K**
Fe-N(1)	1.994(2)	2.203(2)				
Fe-N(2)	1.939(3)	2.104(4)				
Fe(1)-N(1)			1.997(3)	2.002(3)	2.009(3)	2.214(4)
Fe(1)-N(2)			2.008(4)	2.003(3)	2.010(3)	2.221(4)
Fe(1)-N(3)			1.936(4)	1.940(3)	1.954(3)	2.094(5)
Fe(2)-N(4)			2.010(4)	2.109(3)	2.201(3)	2.227(4)
Fe(2)-N(5)			2.002(3)	2.098(3)	2.190(3)	2.210(4)
Fe(2)-N(6)			1.938(4)	2.015(3)	2.082(3)	2.106(5)
N(1)-Fe-N(2)	90.33(6)	90.87(5)				
N(1)-Fe(1)-N(2)			88.14(14)	88.25(10)	88.26(11)	88.14(15)
N(1)-Fe(1)-N(3)			90.2(2)	90.37(12)	90.45(14)	91.3(2)
N(2)-Fe(1)-N(3)			90.5(2)	90.38(12)	90.28(14)	90.2(2)
N(4)-Fe(2)-N(5)			88.55(13)	88.48(9)	88.48(10)	88.52(14)
N(4)-Fe(2)-N(6)			90.3(2)	90.41(11)	90.48(13)	90.6(2)
N(5)-Fe(2)-N(6)			90.1(2)	90.28(11)	90.62(13)	90.9(2)

Table S6. Selected bond lengths [Å] and angles [°] for **1@1.32PhF**, **1@1.14PhF$_2$** and **1@1.8PhF$_3$**.

	**1@1.32PhF**		**1@1.14PhF$_2$**	**1@1.2PhF$_3$**
**T / K**	**130 K**	**260 K**	**140 K**	**150 K**
Fe-N(1)	1.9908(14)	2.208(2)	1.974(2)	1.990(2)
Fe-N(2)	1.930(2)	2.096(4)	1.971(2)	1.989(3)
Fe-N(3)			1.986(2)	1.935(3)
N(1)-Fe-N(2)	90.31(4)	90.90(5)	84.49(8)	88.67(10)
N(1)-Fe-N(3)			87.38(9)	90.21(10)
N(2)-Fe-N(3)			88.28(9)	89.36(11)

Table S7. Selected bond lengths [Å] and angles [°] for **1@2PhF$_n$** (n = 4, 5, 6)

	**1@2PhF$_4$**	**1@2PhF$_5$**		**1@2PhF$_6$**
**T / K**	**150 K**	**120 K**	**260 K**	**120 K**
Fe-N(1)	1.987(2)	1.931(4)	2.180(6)	2.0030(13)
Fe-N(2)	1.998(2)	1.986(4)	2.204(6)	1.9997(13)
Fe-N(3)	1.937(2)	1.985(4)	2.083(8)	1.9390(15)
N(1)-Fe-N(2)	88.58(8)	89.74(15)	88.6(2)	88.13(5)
N(1)-Fe-N(3)	90.35(9)	90.22(15)	91.0(2)	90.96(6)
N(2)-Fe-N(3)	90.28(9)	88.05(15)	88.9(2)	90.14(6)

Figure S4. Supramolecular organization of the guests in the nanochannels running along z-direction for **1@2PhF$_5$** and **1@2PhF$_4$(1,2,3,4) and 1@2PhF$_4$(1,2,4,5)**. The large red-white bar denotes the center of the pore. The thin yellow-red line corresponds to the shortest C···C intermolecular distance smaller than the sum of the van der Waals radii (3.4 Å). Thermal ellipsoids are drawn at 30% probability

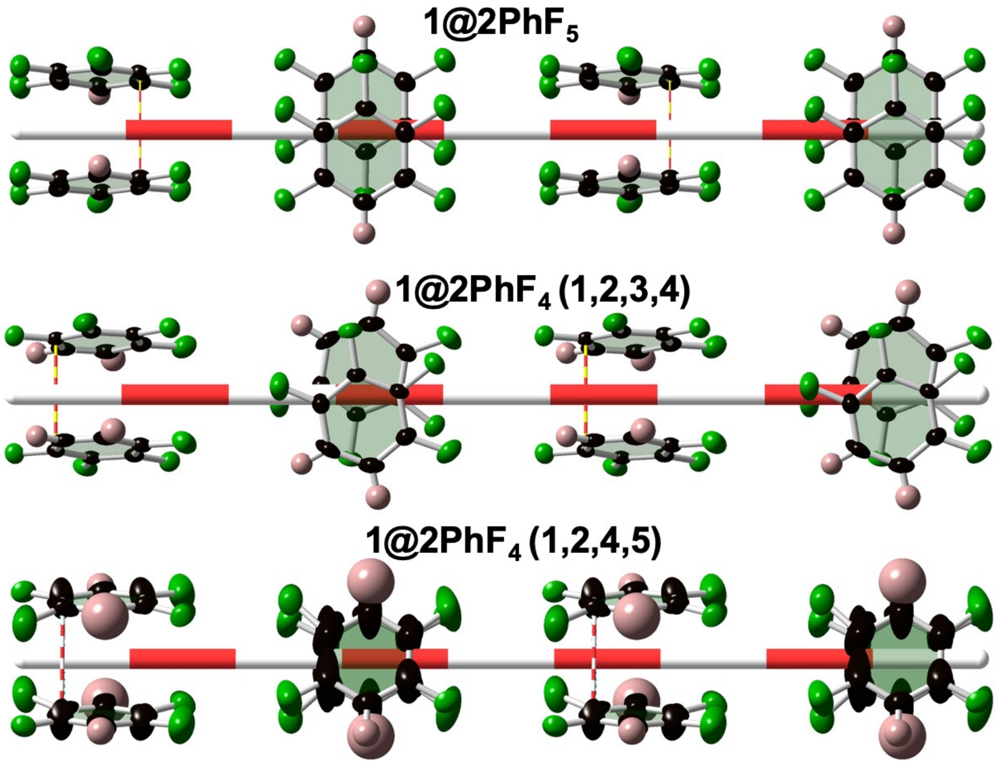

Figure S5. Relevant host-host, host-guest and guest-guest supramolecular interactions for **1@2PhF$_4$(1,2,3,4)** and **1@2PhF$_5$**. Red-white and red-blue bonds represent, respectively, the shortest S···C host-host and C···C guest···guest interactions shorter than the sum of the van der Waals radii.

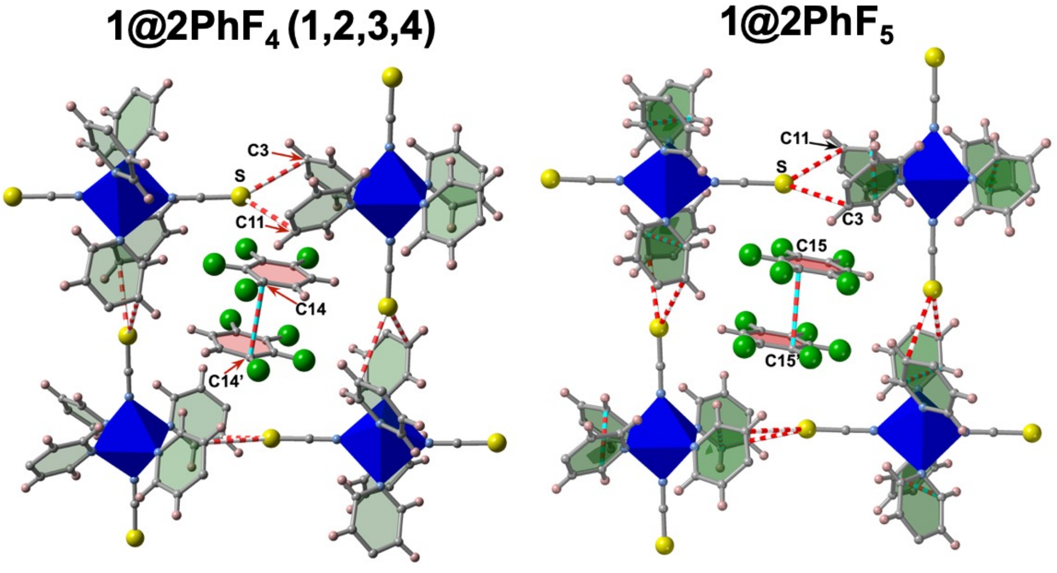

Figure S6. Thermal variation of the most relevant supramolecular contacts for **1@2PhCl$_2$**. Grey dotted bonds represent weakening of the interaction becoming significantly larger than the sum of the corresponding van der Waals radii.

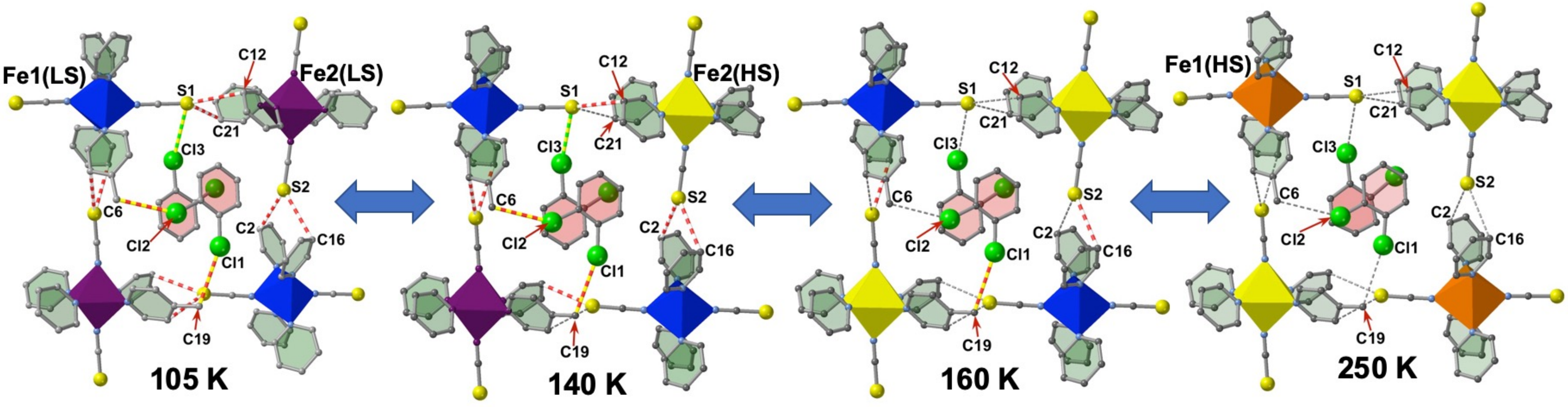

Interaction	105 K	140 K	160 K	250 K
**C2···S2**	3.464(5)	3.480(3)	3.500(4)	3.592(7)
**C2···S16**	3.468(5)	3.480(3)	3.499(4)	3.571(7)
**C12···S1**	3.471(5)	3.495(3)	3.530(4)	3.608(7)
**C21···S1**	3.499(5)	3.531(3)	3.559(4)	3.638(7)
**Cl3···S1**	3.559(2)	3.592(1)	3.626(2)	3.770(4)
**Cl2···C6**	3.379(5)	3.424(4)	3.483(6)	3.666(11)
**Cl1···C19**	3.410(5)	3.430(4)	3.448(5)	3.559(13)

Figure S7. (Left) View of a fragment of the structure of **1@2PhCN** (LS, 120 K) down [001] direction displaying the PhCN rings inside the channels. (Right) Perspective view of the PhCN molecules running parallel to the [001] direction represented by the red-white rod.

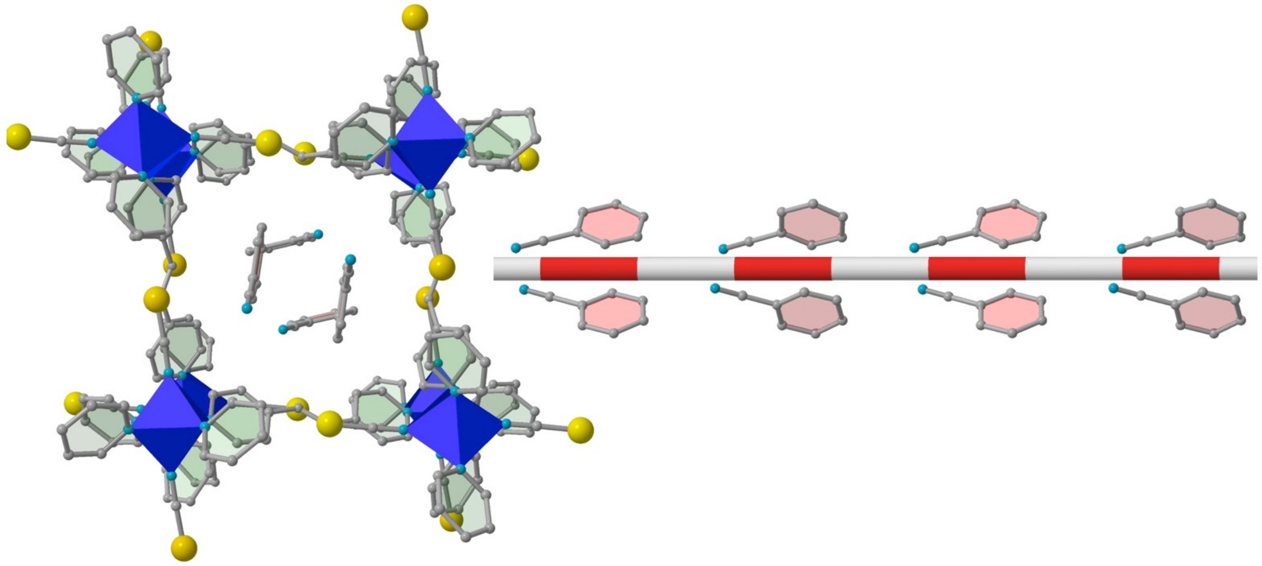

Figure S8. Thermal dependence of $\chi_M T$ for **1@2PhX** (X = F, Cl, Br) emphasizing the subtle differences observed in the low temperature section of the SCO (step and hysteresis).

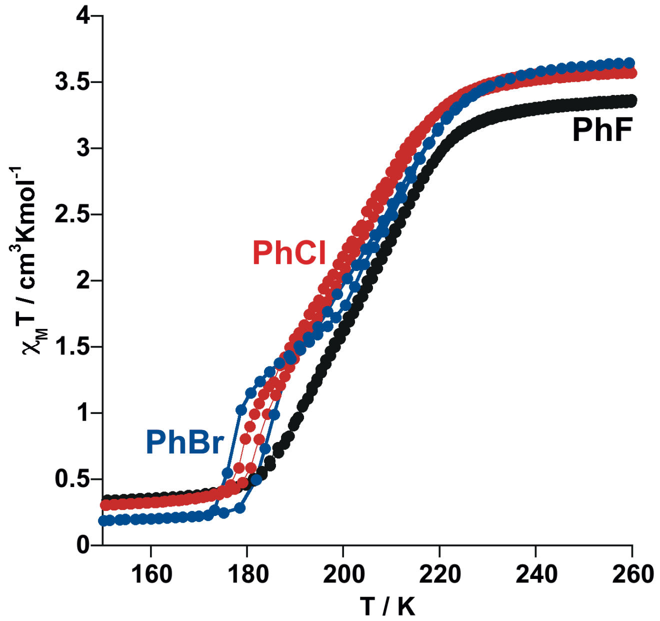

Figure S9. Experimental set up used when the density of the single crystals of the precursor (**1@4($CH_3CN{\cdot}H_2O$)**) and successor (**1@2(PhX_n)**) compounds is smaller than that of the liquid (PhX_n).

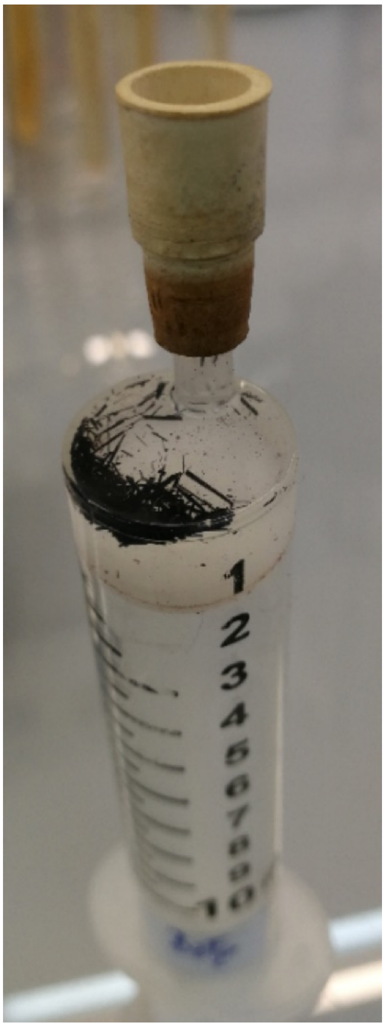

Supplement: Supplementary file 1 — ic2c00014_si_001.pdf [file ic2c00014_si_001.pdf]
